# The Microbe-Derived Short Chain Fatty Acid Butyrate Targets miRNA-Dependent p21 Gene Expression in Human Colon Cancer

**DOI:** 10.1371/journal.pone.0016221

**Published:** 2011-01-20

**Authors:** Shien Hu, Tien Sy Dong, Sushila R. Dalal, Feng Wu, Marc Bissonnette, John H. Kwon, Eugene B. Chang

**Affiliations:** The Martin Boyer Laboratories, Department of Medicine, University of Chicago, Chicago, Illinois, United States of America; University of Barcelona, Spain

## Abstract

Colonic microbiota ferment non-absorbed dietary fiber to produce prodigious amounts of short chain fatty acids (SCFAs) that benefit the host through a myriad of metabolic, trophic, and chemopreventative effects. The chemopreventative effects of the SCFA butyrate are, in part, mediated through induction of p21 gene expression. In this study, we assessed the role of microRNA(miRNA) in butyrate's induction of p21 expression. The expression profiles of miRNAs in HCT-116 cells and in human sporadic colon cancers were assessed by microarray and quantitative PCR. Regulation of p21 gene expression by miR-106b was assessed by 3′ UTR luciferase reporter assays and transfection of specific miRNA mimics. Butyrate changed the expression of 44 miRNAs in HCT-116 cells, many of which were aberrantly expressed in colon cancer tissues. Members of the miR-106b family were decreased in the former and increased in the latter. Butyrate-induced p21 protein expression was dampened by treatment with a miR-106b mimic. Mutated p21 3′UTR-reporter constructs expressed in HCT-116 cells confirmed direct miR-106b targeting. Butyrate decreased HCT-116 proliferation, an effect reversed with the addition of the miR-106b mimic. We conclude that microbe-derived SCFAs regulate host gene expression involved in intestinal homeostasis as well as carcinogenesis through modulation of miRNAs.

## Introduction

Most human sporadic colon cancers develop gradually as accumulating alterations in gene expression transform normal colonic epithelium to adenocarcinoma. This process involves an interplay between genetic and environmental factors, the latter supported by the epidemiological association between increased incidence of colorectal cancers and factors such as increased longevity, exposure to carcinogens, and diets in highly industrialized countries [1]. Among the proposed dietary risk factors is low fiber content, which may lower the bioavailability of short chain fatty acids (SCFAs) that are formed by microbial anaerobic fermentation of dietary fiber [2]. SCFAs such as acetate, proprionate, and butyrate are produced in prodigious amounts and are the most abundant anions in colonic luminal fluid and feces [3]. These microbial products not only provide an important source of energy to the colonic epithelium, but also have widespread trophic effects that include regulation of host genes involved in maintenance of intestinal homeostasis [4].

In undifferentiated, highly proliferative malignant cells, butyrate inhibits proliferation and induces differentiation through a variety of mechanisms including alterations in DNA methylation, selective inhibition of histone phosphorylation and histone deacetylation (HDAC), and modulation of intracellular kinase signaling [5]–[7]. In a human colonic epithelial cell line (HT29), 221 butyrate responsive genes involved in proliferation, differentiation, and apoptosis were identified [6]. Amongst the genes altered by butyrate treatment were many involved in cell cycle regulation, such as the cyclin dependent kinase inhibitor p21, GADD45A, and PTEN [6].

Under normal conditions, proliferation is tightly regulated through the action of cyclins, cyclin dependent kinases (CDKs), and CDK inhibitors which regulate the transitions from G1 to S phase and G2 to mitosis and act as checkpoints to prevent replication if DNA is damaged [8]. In response to signals indicating DNA damage, p21 and p27 bind to cyclin-CDK complexes and induce cell cycle arrest [8], [9]. However, in cancer, this regulated process of cell division and growth is lost. For instance, loss of function of the G1 checkpoint cyclin dependent kinase inhibitor p21 has been linked to carcinogenesis and p21 loss is observed in 79% of colon cancer tumors by immunohistochemistry [10], [11].

Butyrate induces p21 gene transcription via a p53 independent pathway involving non-competitive inhibition of HDAC [12]–[14]. However, the possibility that some of butyrate's actions on p21 gene expression might be mediated through miRNA-dependent translational mechanisms has not been previously explored. HDAC inhibitors have recently been studied as a new group of anti-cancer epigenetic treatment tools, and a HDAC inhibitor, suberoylanilide hydroxamic acid (SAHA), is FDA approved for the treatment of cutaneous T cell lymphoma [15]. Furthermore, HDAC inhibitors have been implicated in miRNA regulation in multiple types of malignancies. Treatment of the breast cancer cell line SKBr3 with the hydroxamic acid HDAC inhibitor LAQ824 led to significant changes in ∼40% of the cell's expressed miRNAs [16]. SAHA treatment of the human lung carcinoma cell line A549 led to significant alterations in the expression of 64 miRNAs [17]. The influence of the HDAC inhibitor and microbial product butyrate on miRNA expression in colon cancer tissues has not been investigated.

miRNAs are ∼22 nucleotide, non-coding RNAs that play an important role in regulating cell proliferation, apoptosis, and differentiation [18]. Greater than 1000 human miRNAs have been identified, and most are believed to target hundreds of genes [19]. Dysregulation of miRNA expression can contribute to carcinogenesis by increasing proto-oncogene expression or down-regulating tumor suppressors [20]. For example, miRNAs regulate many key proteins in the signaling pathways of colorectal cancer, e.g. the miR-106b family reduces p21 expression and affects cell cycle progression [21]–[23]. Amongst the miR-106b predicted targets, silencing of p21 with siRNA most closely phenocopies miR-106b gain of function [22].

In this study, we hypothesized that the anti-cancer effects of the microbe derived SCFA butyrate may be mediated in part via changes in miRNA expression. We performed miRNA microarray studies on human colon cancer HCT-116 cells treated with butyrate and found significant alterations in miRNA profiles, including decreased expression of the miR-106b family. miRNA microarray analysis of sporadic-type human colon cancers found increased expression of the miR-106b family. Butyrate was found to induce p21 expression, which was associated with a significant decrease in cell proliferation. The addition of a miR-106b mimic reversed the increased p21 expression and decreased cell proliferation induced by butyrate. These findings have uncovered a unique mechanism of microbial interaction with host gene expression that involves alterations of miRNA profiles to restrain cell cycling and inhibit colon cancer cell proliferation.

## Materials and Methods

### Ethics Statement

Surgical human colonic biopsies were obtained from colon cancer patients at the University of Chicago Medical Center under a protocol approved by the Institutional Review Board. Written informed consent was obtained prior to the collection of tissue specimens. All clinical investigations using human subjects were conducted according to the principles expressed in the Declaration of Helsinki.

### Cell Culture

Human HCT-116 colon cancer cells were acquired from ATCC. Cells were grown at 37°C in high glucose DMEM medium (Invitrogen) containing 10% (vol/vol) fetal bovine serum, 50 µg/ml L-glutamate, 50 µg/ml streptomycin, and 50 U/ml penicillin. Cells were treated with 1–2 mM butyrate for 24 to 48 hours prior to harvest for each individual assay. Cells were rinsed twice and scraped into ice-cold phosphate buffered saline (PBS), pelleted (14,000 g×20 sec), then lysed for RNA and protein extraction.

### Human colonic biopsies

Surgical human colonic biopsies from tumor tissue and surrounding normal appearing colonic mucosa (at least 5 cm away the tumor border) were obtained by a colorectal surgeon. After removal, biopsies were immediately placed on ice and rinsed in ice-cold PBS prior to cell lysis for RNA extraction.

### miRNA microarray

Total RNA was extracted from HCT-116 cells and human colonic tissue samples using the mirVana™ miRNA Isolation Kit (Ambion) according to the manufacturer's protocol. HCT-116 cell miRNA was analyzed using the miRCURY LNA™ microarray v.11.0 (Exiqon) that contains capture probes targeting all miRNAs for human, mouse, or rat registered in the miRBase version 13 at the Sanger Institute. All samples were pooled to create a common reference. One µg total RNA from each sample and the pooled common reference was labeled using the miRCURY™ LNA Array power labeling kit (Exiqon, Denmark). The Hy3™-labeled samples and a Hy5™-labeled reference RNA sample were mixed pair-wise and hybridized to the miRCURY™ LNA arrays. The microarray slides were scanned using the Agilent G2565BA Microarray Scanner System (Agilent Technologies, Inc., USA) and the image analysis was carried out using the ImaGene 8.0 software (BioDiscovery, Inc., USA). The quantified signals were background corrected (Normexp with offset value 10) and normalized using the global Lowess (LOcally WEighted Scatterplot Smoothing) regression algorithm [24]. The data is expressed as the normalized log2 transformed Hy3/Hy5 ratio.

In a similar fashion, human colonic tissue miRNAs were analyzed using mirVana miRNA Bioarrays v.2 (Ambion), which utilizes version 8.0 of the miRBase sequence database. The samples were labeled with the mirVana miRNA labeling kit and hybridized to the miRNA bioarrays per the manufacturer's instructions. The arrays were scanned using the GenePix4000B in the University of Chicago Functional Genomics Core Facility. A total of 12 miRNA profiles were generated from 6 paired colonic tissue samples.

### Real-time PCR for miRNAs

Total RNA was extracted from pelleted HCT-116 cells by Trizol (Invitrogen, Grand Island, NY) according to the manufacturer's instructions. Complementary DNA was synthesized from total RNA samples extracted from HCT-116 cells or human colon tissues using the NCode™ miRNA First-Strand cDNA Synthesis Kit (Invitrogen). Real-time PCR was performed with an iCycler (Bio-Rad) using the iQSYBR Green PCR supermix (Bio-Rad) with miRNA specific primers and a universal qPCR primer according to the manufacturer's protocol for the NCode Kit (**Table S1**). The two-step quantification cycling protocol (45 cycles of 95°C for 15 seconds and then 60°C for 15 seconds) was used. The Ct value is defined as the cycle number at which the fluorescence crosses a fixed threshold above the baseline. A small nucleolar RNA, RNU48, was measured as endogenous control [25]. For a relative quantification, fold changes were measured using the ΔΔCt method. For each sample, the Ct value of each miRNA was measured and compared to RNU48 as ΔCt, (ΔCt  =  Ct _miRNA_ – Ct _RNU48_). The fold change of miRNA in experimental samples relative to control samples was determined by 2^−ΔΔCT^, where ΔΔCt  =  ΔCt _Unknown_ –ΔCt _Control_ [26]_._

### Real-time PCR for p21 mRNA

Total RNA was extracted from pelleted HCT-116 cells with Trizol. Complementary DNA was synthesized using SuperScript III (Invitrogen) and a random hexonucleotide primer. The sense and antisense primers for p21 (CDKN1A, NM_000389.3) are: 5′-TCACTGTCTTGTACCCTTGTGCTT-3′ and 5′- AGAAATCTGTCATGCTGGTCTGCC-3′; for GAPDH: 5′-GGCAAATTCAACGGCACAGT-3′ and 5′-AGATGGTGATGGGCTTCCC-3′. Real-time PCR was performed with an iCycler using iQSYBR Green PCR supermix (Bio-Rad). For each sample, the Ct value of p21 mRNA was measured and compared to the GAPDH endogenous control as ΔCt, (ΔCt  =  Ct_p21_ – Ct _GAPDH_). The fold change of miRNA in experimental samples relative to control samples was determined by 2^−ΔΔCT^.

### Western Blot

Pelleted HCT-116 cells were homogenized in 10 mM Tris, pH 7.4, 5 mM MgCl_2_, complete protease inhibitor cocktail (Roche Molecular Biochemicals), 50 U/ml DNAse (Amersham), and 50 U/ml RNAse (Ambion). Protein was quantified using the bicinchoninic acid method. Protein was solubolized in 3X Laemmli stop solution by heating to 65°C for 10 minutes.

Twenty microgram protein samples were separated by SDS-PAGE and transferred to polyvinylidene difluoride (PVDF) membranes in 25 mM Tris, pH 8.8; 192 mM glycine; 15% vol/vol methanol. Membranes were blocked with 5% wt/vol non-fat dry milk in tween-tris buffered saline (TTBS). Primary antibodies, specific for p21 (BD Bioscience), Hsc70 (SPA815; Stressgen) and β-actin (Cell Signaling), were added and incubated overnight at 4°C. Membranes were washed with TTBS, incubated with horseradish peroxidase-conjugated species-appropriate secondary antibodies (Jackson Immunoresearch, West Grove, PA) for 1 hour at room temperature, and developed using an enhanced chemiluminescence system (Supersignal; Pierce, Rockford, IL).

Quantification of images was done by scanning densitometry using NIH Image J 1.54 software (National Institutes of Health, Bethesda, Maryland).

### Cell Proliferation Assay

Cell proliferation was measured using the WST-1 reagent (Roche Applied Science) according to the manufacturer's protocol. HCT-116 cells were cultured on a 96 well flat-bottom plate. After reaching 50% confluence, wells were treated with the indicated concentration of butyrate for 24 hrs. Plates were read on a microplate reader at 450 nm before and 45 minutes after adding the WST-1 reagent. The reference wavelength was 650 nm. Cell proliferation rates were calculated according to the manufacturer's protocol.

### Cell transfection with miRNA

TransIT-LT1 (Mirus, WI) transfection reagent was used to transfect HCT-116 cells with an engineered miR-106b (Ambion's Pre-mir MiRNA Precursor Molecules) according to the manufacturer's protocol. A control miRNA (miR-C), with identical GC content but no sequence homology to miR-106b, was used as a control. Cells were transfected for 48 hours prior to harvest.

### Luciferase Reporter Assays

Modified pGL3 constructs with the p21 3′UTR downstream of the firefly luciferase coding sequence were a generous gift from Dr. V. Narry Kim of the Department of Biological Sciences, Seoul National University [27]. Six hours after butyrate treatment, HCT-116 cells were transiently transfected with modified pGL3 constructs and pRL-TK plasmids (Renilla luciferase driven by thymidine kinase promoter, E2241, Promega) using the TransIT LT-1 transfection reagent. Cells were harvested by shaking in 500 µl lysis buffer (Promega). Firefly and *Renilla* luciferase activities in the lysate were determined in triplicate with a Dual-Luciferase Reporter assay system, according to the manufacturer's instructions (Promega). Firefly luciferase activity was normalized to *Renilla* luciferase activity.

### Statistical Analysis

Results are presented as mean ±SEM for the indicated number of experiments. The results of multiple experiments were analyzed by student's t-test or ANOVA using Bonferroni correction for multiple comparisons.

For the miRNA arrays, a two-tailed T-test calculated between the sample and reference groups identified miRNAs with p-values lower than 0.05. These miRNAs were then chosen for further study with RT-PCR.

## Results

### Butyrate alters miRNA expression in human colon cancer HCT-116 cells, including members of the miR-106b family

**Figure 1:**
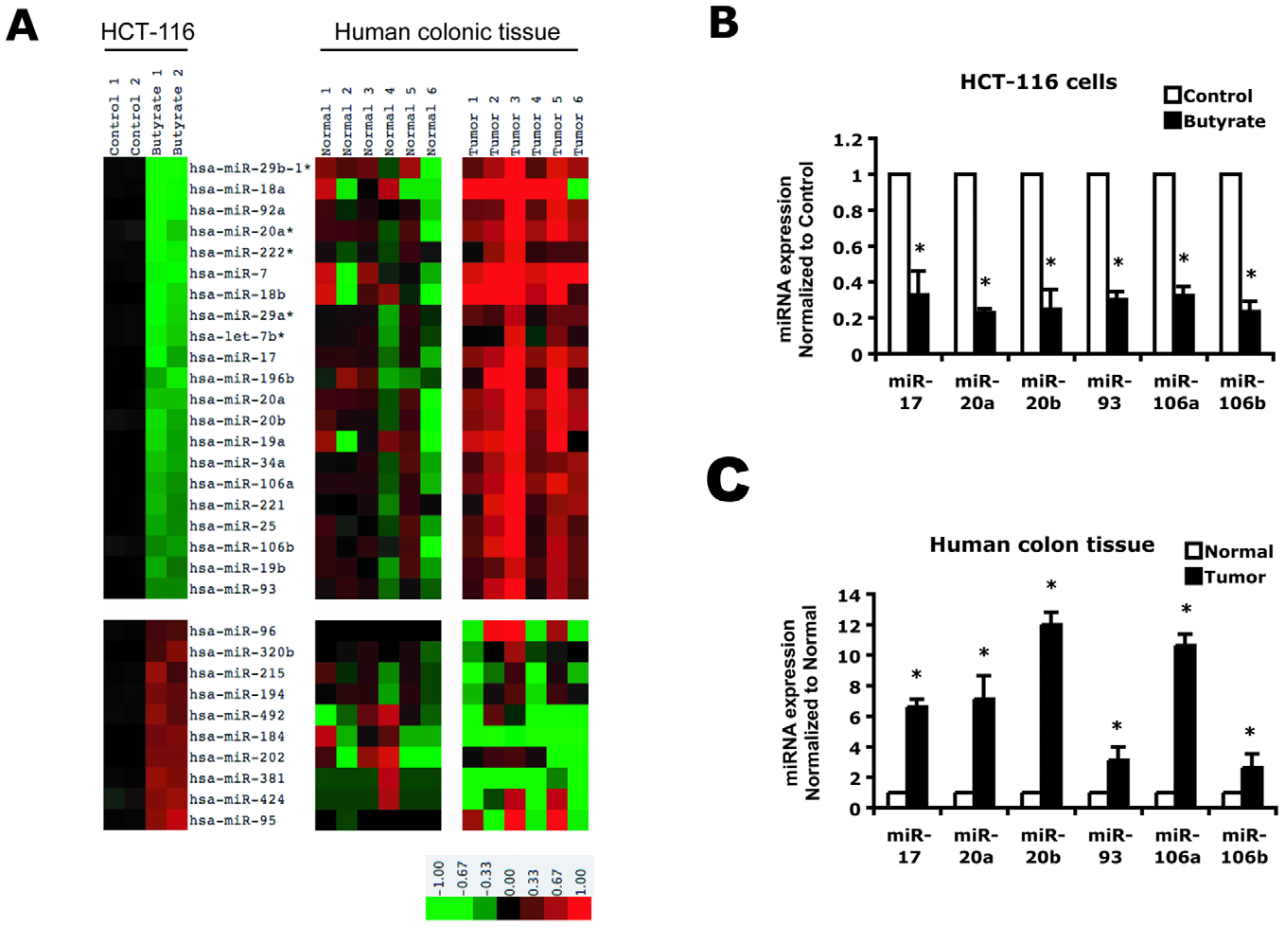
Butyrate treatment of HCT116 cells alters expression of miRNAs aberrantly expressed in human colon cancers. A) miRNA microarray profiles were performed on RNA extracted from HCT-116 cells treated with vehicle or 1 mM butyrate and human colon tissues from patients with sporadic colonic cancer and adjacent normal-appearing tissue. Data was normalized using the global Lowess regression algorithm and is expressed as log base 2 transformed ratios of the sample signal to the control reference pool signal. Heat maps are represented. The red color in the heat map represents increased expression as compared to the pooled reference control, and green represents decreased expression as compared to the pooled control. Changes in expression of the miR-17 – 106b family were confirmed by real-time PCR in B) HCT-116 cells and C) human colon tissues. Results are means ± SE, n = 4. * indicates p<0.05 for sample as compared to control.

To study the effects of butyrate on miRNA expression in colonic cancer cells, expression profiles of miRNAs in butyrate-treated HCT-116 cells were measured using a microarray. Vehicle-treated HCT-116 cells were analyzed as a control. Forty-four miRNAs demonstrated significant changes in expression in response to butyrate treatment. The changes in expression of 13 of the 26 miRNAs that decreased and 5 out of the 18 miRNAs that increased were confirmed using real-time, quantitative PCR (**Figure S1**). Thirty-one of the 44 miRNAs with changes in expression are shown in the heat map on the left panel of **Figure 1A**. Multiple members of the miR-17-92a, miR-18b-106a, and miR-25-106b clusters were significantly decreased in response to butyrate.

Expression levels of these miRNAs were also assessed in human sporadic colon cancers and surrounding normal appearing colon by microarray and are shown in the right panel of **Figure 1A** **.** The miRNAs decreased in butyrate-treated HCT-116 cells were dramatically increased in tumor tissues as compared to normal controls. miRs-17, -20a, -20b, -93, -106a and -106b were all decreased in response to butyrate treatment. These miRNAs share the same seed region sequence and thus target the same binding sites in the 3′UTRs of target mRNAs. Using real time PCR, we confirmed that butyrate down-regulated these miRNAs in HCT-116 cells **(** **Figure 1B**). We also confirmed that these miRNAs were highly over-expressed in human colon cancer samples (**Figure 1C**), suggesting that some anti-cancer effects of butyrate are mediated by suppressing the miRNAs that are upregulated in colon cancer.

### miR-106b inhibits butyrate-induced p21 protein expression

**Figure 2:**
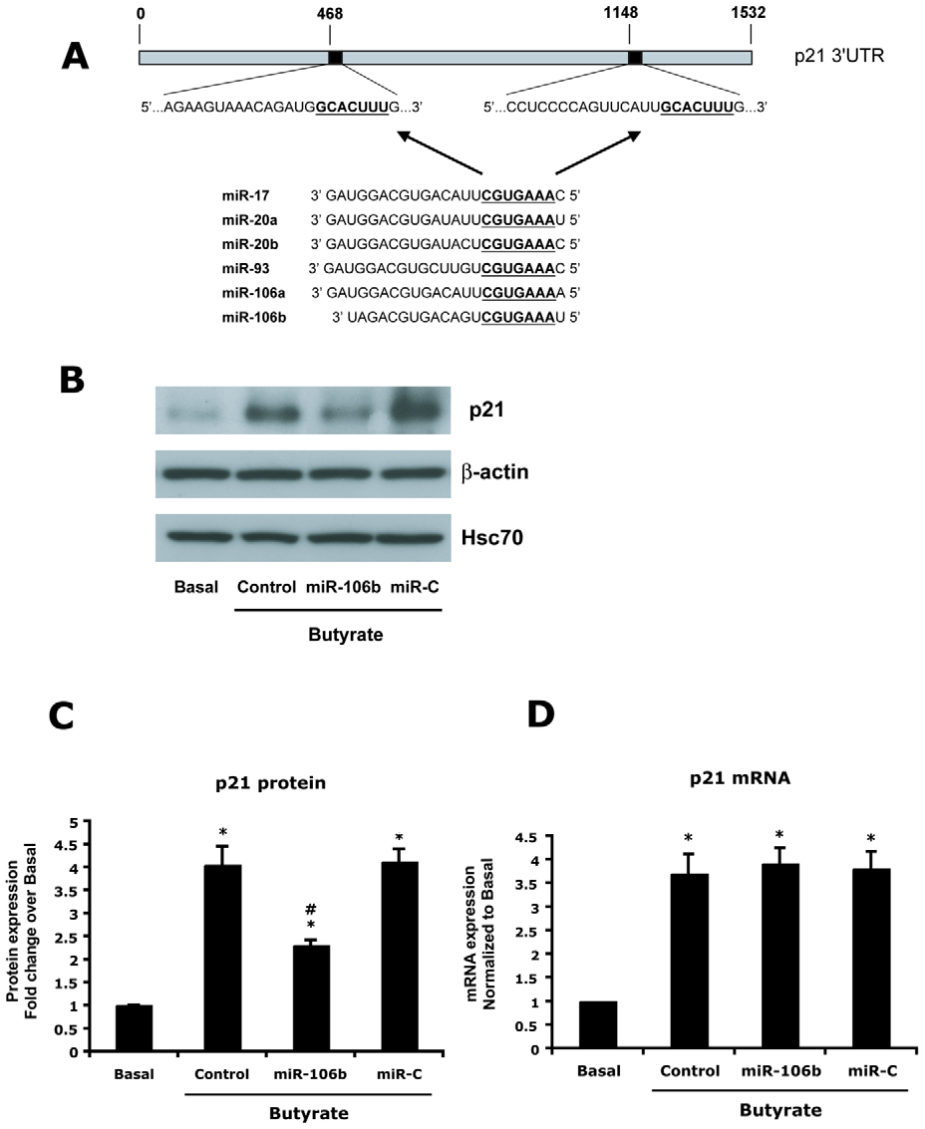
Butyrate-induced p21 protein expression is inhibited by miR-106b. A) Schematic of common seed regions in the miR-17 – 106b family that target the p21 3′UTR at two sites. HCT-116 cells were treated with butyrate (2 mM) or vehicle and were transfected with miR-106b mimic or control miRNA (miR-C) for 24 hours prior to harvest. B) Western blots for p21, β-actin and Hsc70 are shown, and are representative of 4 individual experiments all with similar results. C) Densitometry results of Western blots of p21 normalized to β-actin. D) p21 mRNA abundance was analyzed by real-time PCR. Results are means ± SE, n = 4. * indicates p<0.05 compared to basal. # indicates p<0.05 compared to control.

Prior studies showed that the 3′ UTR of p21, which regulates cancer cell proliferation, contains two binding sites for the miR-17 – 106b seed sequence (**Figure 2A**) and is inhibited by these miRNAs in various types of cancer [reviewed in 13–15, 22]. We, therefore, analyzed the effects of butyrate and a miR-106b mimic on p21 mRNA and protein levels in HCT-116 cells. As shown in **Figure 2B and 2C**, butyrate increased p21 protein expression four fold after 24 hours of treatment. An exogenous miR-106b mimic dampened butyrate-induced p21 protein expression as compared with cells treated with butyrate alone whereas control miRNA molecules had no effect on p21 expression.

To determine the effect of butyrate and miRNAs on p21 gene expression, cellular p21 mRNA levels were measured by real-time PCR (**Figure 2D**). Butyrate induced a 3.6-fold increase in p21 mRNA abundancy. In contrast, the miR-106b mimic had no effect on p21 mRNA expression.

### The 3′UTR of p21 mediates translational regulation by butyrate and miR-106b

**Figure 3:**
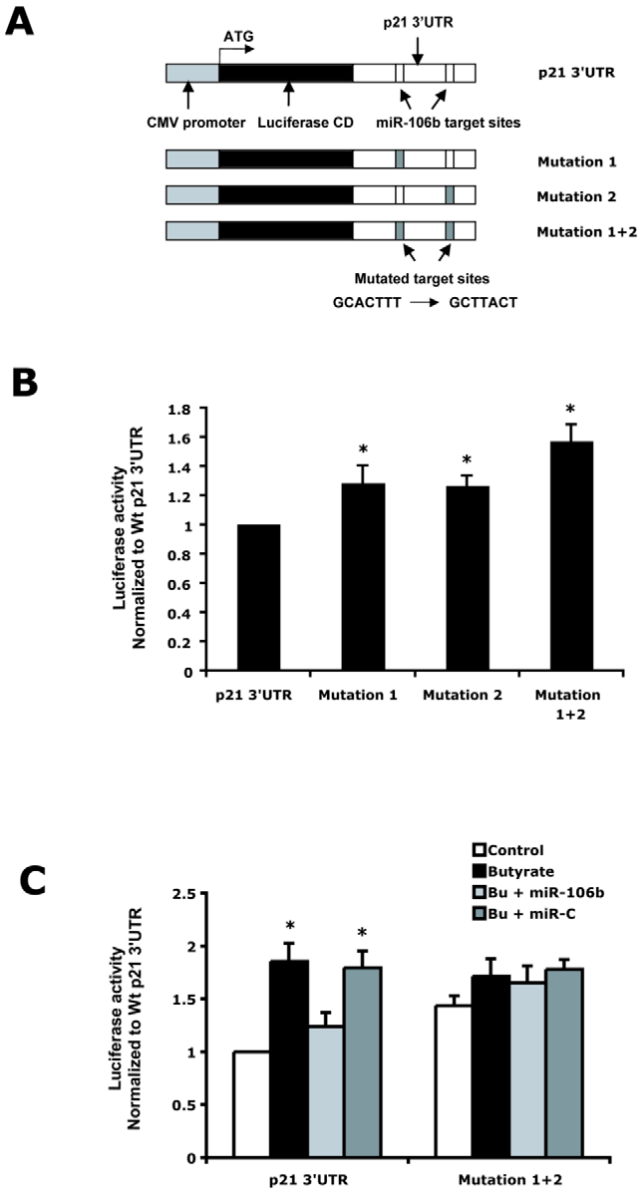
miR-106b regulates p21 translation via two target sites in the 3′UTR. A) Schematic of the luciferase reporter constructs containing the p21 mRNA 3′UTR, which includes two binding sites for the miR-17-106b family. Mutations were generated in these miR-106b target sites. HCT-116 cells were transiently co-transfected with firefly luciferase pGL3 vectors containing the wild type or mutated p21 3′UTR and pRL-TK Renilla luciferase control vector. Cells were also treated with butyrate (2 mM) or vehicle and miR-106b mimic or control miRNA molecules (miR-C). Cells were harvested for luminescence measurement 48 hrs after transfection. B) Basal luciferase expression of reporter constructs with wild type or mutated p21 3′UTR. * indicates p<0.05 compared to p21 3′UTR C) Luciferase expression in cells after butyrate and miRNA treatment. * indicates p<0.05 compared to control. Results are means ± SE, n = 4.

To investigate the effect of miR-106b on translational regulation of p21 expression, HCT-116 cells were transiently transfected with modified luciferase reporter vectors containing either the wild type p21 3′UTR or p21 3′UTRs containing mutations in either one or both of the miR-106b binding sites (**Figure 3A**). Firefly luciferase expression was used to assess cis regulation via the p21 3′ UTR. The pRL-TK vector expressing Renilla luciferase was co-transfected to control for transfection efficiency. Under basal conditions, mutations in the individual miR-106b target regions in the p21 3′UTR at nucleotides 468-474 and nucleotides 1148-1154 resulted in a 28% and 26% increase in luciferase activity respectively (**Figure 3B**). Furthermore, mutations in both miR-106b target regions resulted in a 57% increase in luciferase activity, suggesting that both binding sites mediate miRNA inhibition of basal p21 expression in cancer cells.

Butyrate stimulated luciferase activity 85% over control in HCT-116 cells transfected with the luciferase containing p21 3′ UTR vector (**Figure 3C**). Butyrate's effects on luciferase expression were reversed by the addition of exogenous miR-106b mimics, but not control miRNA molecules (miR-C). Furthermore, the luciferase activity of HCT-116 cells transfected with the chimeric vector containing both mutant miR-106b target sites was not altered with butyrate treatment or with butyrate in the presence of exogenous miR-106b.

### Butyrate's effect on cell proliferation is inhibited by miR-106b

**Figure 4:**
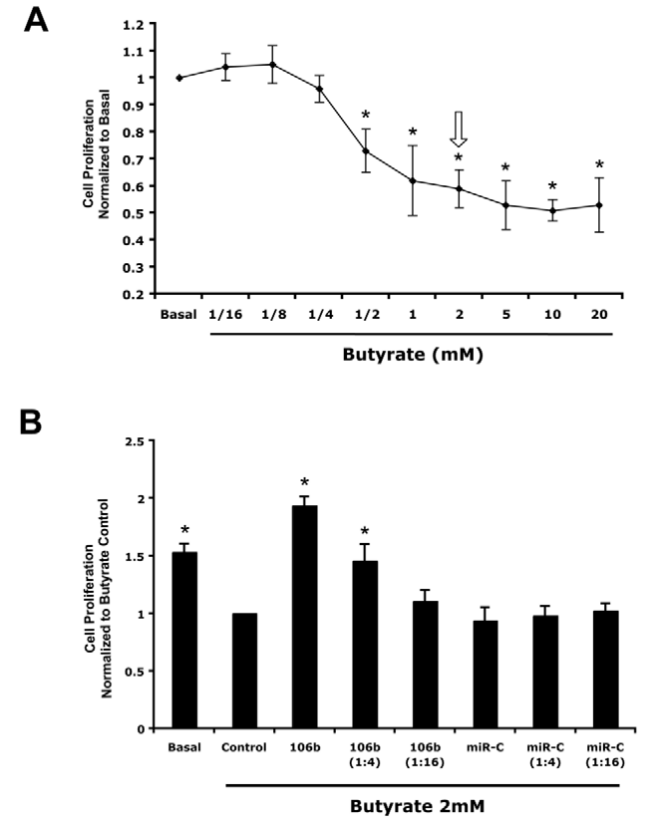
miR-106b reverses butyrate's anti-proliferative effects. HCT-116 cell proliferation rate was measured with the WST-1 proliferation kit. A) HCT-116 cells were treated with the indicated concentration of butyrate or vehicle for 24 hrs before WST-1 measurement. * indicates p<0.05, compared to basal B) HCT-116 cells were transfected with the miR-106b mimic or control miRNA (miR-C) with the indicated concentrations immediately before treatment with 2 mM butyrate. Cells treated only with butyrate were analyzed as Control. * indicates p<0.05 compared to butyrate alone. Results are means ± SE, n = 5.

Since p21 inhibits cell cycle progression, we examined the role of miR-106b on butyrate's anti-proliferative effects. HCT-116 cells were treated with multiple dilutions of butyrate for 24 hours and a WST-1 proliferation assay was performed. As shown in **Figure 4A**, butyrate dose-dependently inhibited cell proliferation. The anti-proliferative butyrate concentrations were in the physiological range of 0.5 to 20 mM [28], [29]. Cells transfected with exogenous miR-106b or control for 24 hrs prior to 2 mM butyrate exposure were also analyzed (**Figure 4B**). The butyrate-induced inhibition of cell proliferation was reversed by the addition of miR-106b mimic molecules in a dose-dependant manner (**Figure 4B**). In contrast, control miRNA molecules (miR-C) showed no effect on the butyrate-induced inhibition of cell proliferation.

## Discussion

**Figure 5:**
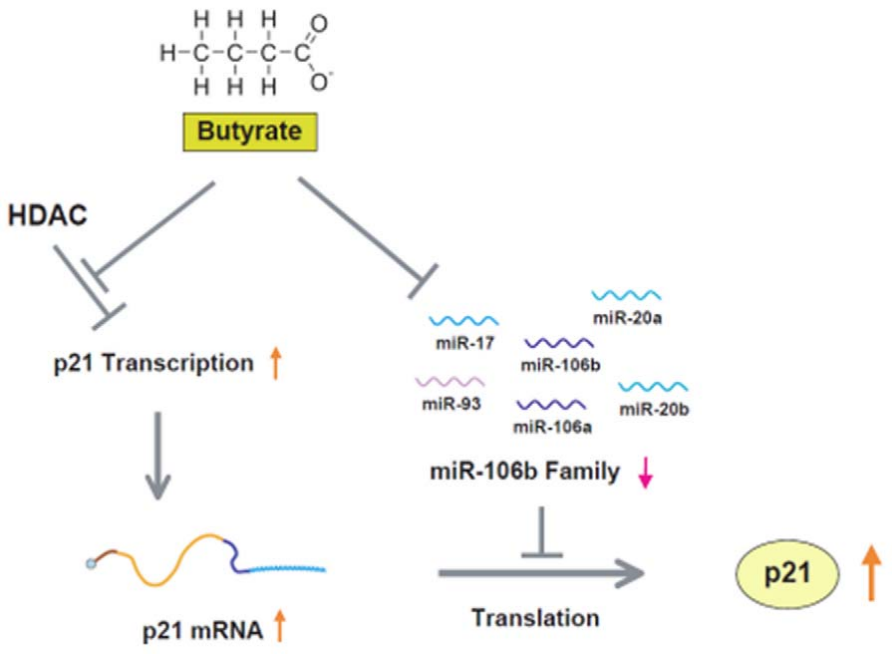
Butyrate regulates p21 expression via HDAC inhibition and decreased expression of the miR-106b family. Butyrate inhibits histone deactylases (HDAC), allowing increased histone acetylation, decreased higher order chromatin folding, and increased transcription of p21. Butyrate also decreases the expression of miR-106b, and several other miRNAs with the same seed sequence region. The miR-106b family inhibits p21 translation, and therefore decreased expression of the miR-106b family leads to increased p21 translation.

In this study, we identify, for the first time, an important growth regulatory role for colonic epithelial miRNAs in mediating the effects of the microbe-derived short chain fatty acid butyrate on host gene expression. Interestingly, in HCT-116 cells, butyrate suppressed many of the same miRNAs increased in human colon cancers. One of these miRNAs, miR-106b, was found to target p21. Butyrate and miR-106b treatment of a p21 3′UTR luciferase reporter construct in HCT116 cells indicates that butyrate-stimulated p21 expression is translationally inhibited in part by miR-106b. This partial inhibition by miR-106b confirms previous reports that butyrate also regulates p21 expression via a miRNA independent mechanism, through its inhibition of HDAC [7], [13], [14]. We propose that the microbial product butyrate regulates the cell cycle through both epigenetic and translational regulation through its dual role as a HDAC inhibitor and inhibitor of miR-106b expression (**Figure 5**).

These results have important implications for intestinal homeostasis and carcinogenesis. These data would suggest that these miRNAs play a role in colonic carcinogenesis and that their reduction by butyrate is an important mechanism of its anti-cancer effects. Six of these miRNAs are in the same miRNA family (miR-17, miR-20a, miR-20b, miR-93, miR-106a, and miR-106b), share an identical seed sequence, and thus target the same binding sites in the 3′UTRs of target mRNAs. Therefore, suppression of their target genes during the carcinogenic process might represent a patterned cell response to promote cell proliferation and/or maintenance of the undifferentiated state.

Because many miRNAs are also located in intronic regions of encoding genes, the miRNA response is likely coordinated with transciptionally activated genes that contribute to the overall process of carcinogenesis. As an example pertinent to our findings, the miR-106b-25 polycistron is located within intron 13 of the MCM7 gene on chromosome 7q22.1 [23]. MCM7 (minichromosome maintenance protein 7) regulates DNA replication during the S phase. In quiescent cells, human MCM7 mRNA levels are almost undetectable, but its expression is induced as cells enter the cell cycle. The MCM7 promoter has three E2F sites, three GC boxes, and an E box [30]. In hepatocellular carcinoma (HCC), the expression of the miR-106b precursor strongly correlates with MCM7 expression, indicating that the miR-106b-25 polycistron is coordinately transcribed under the influence of the MCM7 promoter. High levels of expression of the transcription factor E2F1 in HCC also correlated with increased miR-106b expression [31]. In gastric cancer cells, E2F1 also appears to regulate miR-106b-25 expression in parallel with increases in MCM7 expression [32]. In mouse fibroblasts transformed with the EIA and Ras oncogenes, butyrate decreases E2F1 transcripts and protein as well as promoter activation [33]. Thus, butyrate might exert its effect on miR-106b expression via decreased E2F1 expression, though further studies in HCT-116 cells are needed to examine this possibility.

As stated previously, dysregulation of miRNA expression can influence carcinogenesis when the miRNA targets are tumor suppressors or oncogenes. While treatment with miR-106b leads to decreased p21 protein expression, p21 mRNA levels do not change, which is consistent with prior reports that miR-106b regulates p21 through translational inhibition rather than mRNA stability [32]. Although miR-106b regulation of p21 expression has been described in many cell types, there is conflicting data on the mechanism of this interaction. In human mammary epithelial cells and normal lung fibroblasts, miR-106b decreased the p21 mRNA by about 40% [22]. In contrast, in HCC, p21 expression showed no correlation with the expression of the miR106b-25 cluster, and did not seem to be a target of these miRNAs in this cell type [31]. In a human gastric carcinoma derived cell line, miR-106b repressed p21 protein expression, but did not cause a significant change in p21 mRNA levels [32].

In summary, we have discovered a novel mechanism of action for butyrate's anti-cancer effects involving modulation of miRNA profiles and translation-dependent gene expression. As one example, butyrate induces expression of p21, a key regulatory molecule of cell cycle arrest, by suppressing members of the miR-106b family. Butyrate inhibition of miR-106b is also associated with a significant decrease in cancer cell proliferation rates. The latter is reversed by the addition of miR-106b mimics. These findings have uncovered a unique example of microbial regulation of host gene expression that retards cell cycling and inhibits colon cancer cell proliferation.

## Supporting Information

Figure S1 **Butyrate significantly alters the expression of forty-four miRNAs in HCT-116 cells. HCT-116 cells were treated with 1 mM butyrate for 24 hrs.** Isolated total RNA was subjected to miRNA array hybridization. Forty-four miRNAs demonstrated significant changes in expression in response to butyrate treatment. Microarray data was normalized using the global Lowess regression algorithm and is expressed as log base 2 transformed ratios of the sample signal to the control reference pool signal. The changes in miRNA expression were confirmed using real-time, quantitative PCR for 13 of the 26 miRNAs that decreased and 5 of the 18 miRNA s that increased.
(TIF)

Table S1 **Primers Used for Quantitative Real-Time Polymerase Chain Reactions.** Complementary DNA was synthesized from total RNA samples extracted from HCT-116 cells or human colon tissues using the NCode™ miRNA First-Strand cDNA Synthesis Kit (Invitrogen). Real-time PCR was performed with an iCycler (Bio-Rad) using the iQSYBR Green PCR supermix (Bio-Rad) with miRNA specific primers consisting of the entire sequence of the miRNA of interest and a universal qPCR primer according to the manufacturer's protocol for the NCode Kit.
(DOC)
