# Supplementary figures and images for: The Microbe-Derived Short Chain Fatty Acid Butyrate Targets miRNA-Dependent p21 Gene Expression in Human Colon Cancer
Source: PLoS One. 2011 Jan 20;6(1):e16221. doi: 10.1371/journal.pone.0016221 (PMC3024403; doi:10.1371/journal.pone.0016221)

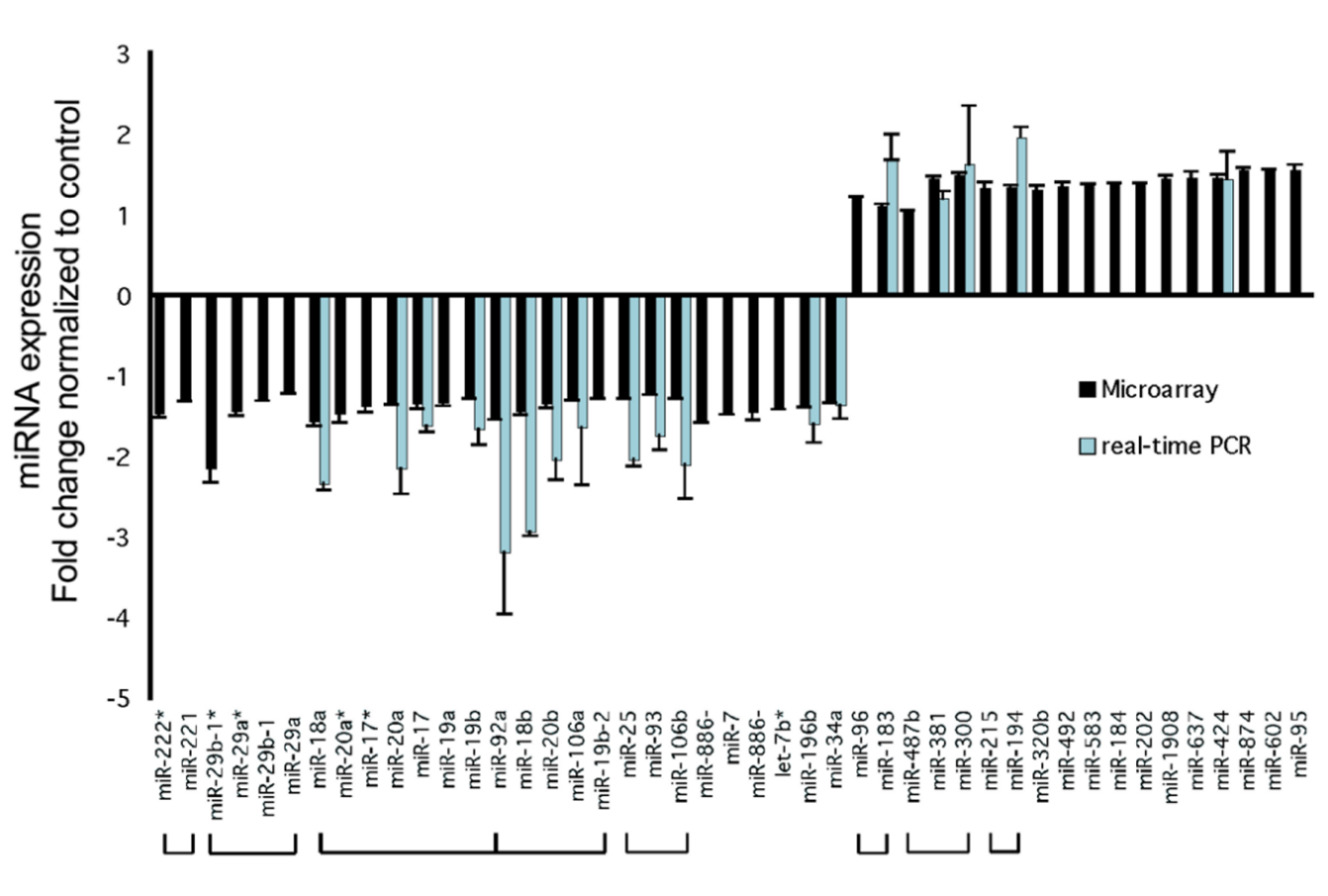

Supplement: Figure S1 — Butyrate significantly alters the expression of forty-four miRNAs in HCT-116 cells. HCT-116 cells were treated with 1 mM butyrate for 24 hrs. Isolated total RNA was subjected to miRNA array hybridization. Forty-four miRNAs demonstrated significant changes in expression in response to butyrate treatment. Microarray data was normalized using the global Lowess regression algorithm and is expressed as log base 2 transformed ratios of the sample signal to the control reference pool signal. The changes in miRNA expression were confirmed using real-time, quantitative PCR for 13 of the 26 miRNAs that decreased and 5 of the 18 miRNA s that increased. (TIF) [file pone.0016221.s001.tif]
