# Supplementary material for: The Microbe-Derived Short Chain Fatty Acid Butyrate Targets miRNA-Dependent p21 Gene Expression in Human Colon Cancer
Source: PLoS One. 2011 Jan 20;6(1):e16221. doi: 10.1371/journal.pone.0016221 (PMC3024403; doi:10.1371/journal.pone.0016221)
Supplement: Table S1 — Primers Used for Quantitative Real-Time Polymerase Chain Reactions. Complementary DNA was synthesized from total RNA samples extracted from HCT-116 cells or human colon tissues using the NCode™ miRNA First-Strand cDNA Synthesis Kit (Invitrogen). Real-time PCR was performed with an iCycler (Bio-Rad) using the iQSYBR Green PCR supermix (Bio-Rad) with miRNA specific primers consisting of the entire sequence of the miRNA of interest and a universal qPCR primer according to the manufacturer's protocol for the NCode Kit. (DOC) [file pone.0016221.s002.doc]

Table S1: Primers Used for Quantitative Real-Time Polymerase Chain Reactions

| Name |  | Primer (5’-3’) |
| --- | --- | --- |
| microRNA universal quantitative PCR primer | Reverse | NCode miRNA First-strand cDNA synthesis kits (Invitrogen) |
| miR-18a | Forward | TAAGGTGCATCTAGTGCAGATAG |
| miR-17 | Forward | CAAAGTGCTTACAGTGCAGGTAGT |
| miR-19b | Forward | TGTGCAAATCCATGCAAAACTGA |
| miR-92a | Forward | TATTGCACTTGTCCCGGCCTGT |
| miR-18b | Forward | TAAGGTGCATCTAGTGCAGTTAG |
| miR-20b | Forward | CAA AGTGCTCATAGTGCAGGTAG |
| miR-106a | Forward | AAAAGTGCTTACAGTGCAGGTAG |
| miR-25 | Forward | CATTGCACTTGTCTCGGTCTGA |
| miR-106b | Forward | TAAAGTGCTGACAGTGCAGAT |
| miR-196 | Forward | TAGGTAGTTTCCTGTTGGG |
| miR-34a | Forward | TGGCAGTGTCTTAGCTGGTTGT |
| miR-183 | Forward | TATGGCACTGGTAGAATTCACT |
| miR-381 | Forward | TATACAAGGGCAAGTTCTCTGT |
| miR-300 | Forward | TATACAAGGGCAGACTCTCTCT |
| miR-194 | Forward | TGTAACAGCAACTCCATGTGGA |
| miR-424 | Forward | CAGCAGCAATTCATGTTTTGAA |
